# Cloning, Expression and Characterization of a Novel Cold-adapted β-galactosidase from the Deep-sea Bacterium *Alteromonas* sp. ML52

**DOI:** 10.3390/md16120469

**Published:** 2018-11-27

**Authors:** Jingjing Sun, Congyu Yao, Wei Wang, Zhiwei Zhuang, Junzhong Liu, Fangqun Dai, Jianhua Hao

**Affiliations:** 1 Key Laboratory of Sustainable Development of Polar Fishery, Ministry of Agriculture and Rural Affairs, Yellow Sea Fisheries Research Institute, Chinese Academy of Fishery Sciences, Qingdao 266071, China; yaocongyv@foxmail.com (C.Y.); weiwang@ysfri.ac.cn (W.W.); qdjz99@163.com (J.L.); dai@ysfri.ac.cn (F.D.); 2 Laboratory for Marine Drugs and Bioproducts, Laboratory for Marine Fisheries Science and Food Production Processes, Qingdao National Laboratory for Marine Science and Technology, Qingdao 266071, China; 3 College of Food Science and Technology, Shanghai Ocean University, Shanghai 201306, China; 4 New Hope Liuhe Co. Ltd., Qingdao 266071, China; zzw19680@163.com; 5 Jiangsu Collaborative Innovation Center for Exploitation and Utilization of Marine Biological Resource, Lianyungang 222005, China

**Keywords:** *Alteromonas*, deep sea, cold-adapted enzyme, β-galactosidase, lactose-free milk

## Abstract

The bacterium *Alteromonas* sp. ML52, isolated from deep-sea water, was found to synthesize an intracellular cold-adapted β-galactosidase. A novel β-galactosidase gene from strain ML52, encoding 1058 amino acids residues, was cloned and expressed in *Escherichia coli*. The enzyme belongs to glycoside hydrolase family 2 and is active as a homotetrameric protein. The recombinant enzyme had maximum activity at 35 °C and pH 8 with a low thermal stability over 30 °C. The enzyme also exhibited a *K*_m_ of 0.14 mM, a *V*_max_ of 464.7 U/mg and a *k*_cat_ of 3688.1 S^−1^ at 35 °C with 2-nitrophenyl-β-d-galactopyranoside as a substrate. Hydrolysis of lactose assay, performed using milk, indicated that over 90% lactose in milk was hydrolyzed after incubation for 5 h at 25 °C or 24 h at 4 °C and 10 °C, respectively. These properties suggest that recombinant *Alteromonas* sp. ML52 β-galactosidase is a potential biocatalyst for the lactose-reduced dairy industry.

## 1. Introduction

Beta-galactosidase (EC 3.2.1.23), a glycoside hydrolase enzyme, catalyzes the hydrolysis of terminal non-reducing β-d-galactose residues into β-d-galactosides and also catalyzes transgalactosylation reactions [1,2,3]. Beta-galactosidases exist naturally in many organisms, including microorganisms, plants and animals [4,5]. Most industrial β-galactosidases are obtained from microorganisms. For example, the enzymes isolated from bacteria [6] and yeast [7], with neutral optimum pH, were used in milk products, and fungal [8] enzymes with an acid optimum pH were used in acid whey products. The main application of β-galactosidase is to hydrolyze lactose in milk in the dairy industry to provide lactose-free milk for lactose-intolerant consumers [9]. Another application of β-galactosidase is to transfer lactose and monosaccharide to a series of galacto-oligosaccharides (GOS) which are functional galactosylated products [10,11,12]. However, β-galactosidase catalyzed at moderate temperatures may cause some issues, e.g., increased production costs, wasted energy and producing undesirable microbial contamination [13]. Cold-adapted β-galactosidases, with low optimum temperatures, could catalyze hydrolysis or transgalactosylation reactions at refrigerating temperatures (4–10 °C), thus potentially overcoming these shortcomings. It may be especially beneficial to the dairy industry which could improve the hydrolysis of lactose at low temperatures.

While a minority of β-galactosidases from fungus are secreted to the extracellular medium, e.g., an acid β-galactosidase from *Aspergillus* spp. [14], β-galactosidases are generally intracellular enzymes in yeast and bacteria. Most reported β-galactosidases are recombinant enzymes derived from heterologous expression than from a natural source. In recent years, the number of cold-adapted β-galactosidases were isolated from psychrophilic and psychrotrophic microorganisms obtained from isothermal cold environments such as polar [15,16,17], deep-sea [18] and high mountainous regions [19]. The main source of enzymes has been obtained from bacterial strains such as *Arthrobacter psychrolactophilus* strain F2 [20], *Arthrobacter* sp. 32c [21], *Halomonas* sp. S62 [22], *Paracoccus* sp. 32d [23], *Pseudoalteromonas haloplanktis* [24] and *Rahnella* sp. R3 [19]. Only a few cold-adapted β-galactosidases have been discovered from other sources, including psychrophilic-basidiomycetous yeast *Guehomyces pullulan* [25] and Antarctic haloarchaeon *Halorubrum lacusprofundi* [26]. The β-galactosidase from *Arthrobacter psychrolactophilus* strain F2 showed the lowest optimum temperature at 10 °C with an optimum pH of 8.

Based on the specific features of sequence, structure, substrate specificity and reaction mechanism, β-galactosidases have been classified into GH1, GH2, GH35 and GH42 families [27]. Most reported microorganism β-galactosidases belong to the GH2 [15,20,23,24,28] and GH42 [19,21,29] families. A typical GH2 β-galactosidase from *E. coli* is made up of five sequential domains and forms a functional tetramer [30]. Most of the characterized cold-adapted β-galactosidases from the GH2 family are tetrameric enzymes, except for a dimeric enzyme from *Paracoccus* sp. 32d and a hexameric enzyme from *Arthrobacter* sp. C2-2 [31]. Hitherto, three crystal structures of cold-adapted β-galactosidases have been obtained: GH42 β-galactosidase from *Planococcus* sp. L4 [32] and two GH2 β-galactosidases from Antarctic bacteria *Arthrobacter* sp. C2-2 [31] and *Paracoccus* sp. 32d [33].

In this study, we report on a gene of β-galactosidase from the marine bacterium *Alteromonas* sp. ML52, isolated from a deep-sea sample. This novel cold-adapted β-galactosidase belongs to the GH2 family and was overexpressed and characterized.

## 2. Results

### 2.1. Characterization and Identification of Strain ML52

**Figure 1:**
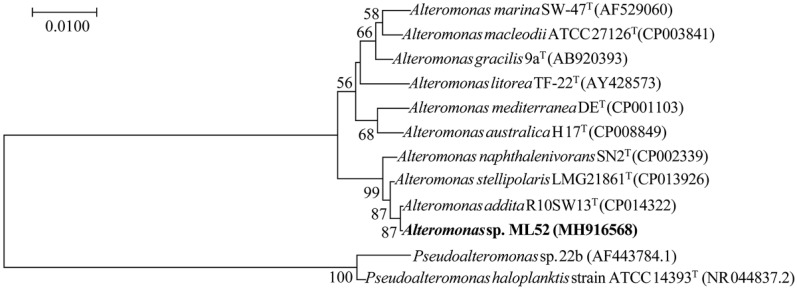
Neighbor-joining tree based on 16S rRNA gene sequences showing the phylogenetic position of strain ML52 and closely related *Alteromonas* and *Pseudoalteromonas* species. Bootstrap values (>50%) were calculated for 1000 replicates. The reference 16S rDNA sequences were collected from EzTaxon-e server (www.bacterio.net/) and the National Center for Biotechnology Information (NCBI) Database and aligned using the ClustalX 2.1 program (Conway Institute UCD Dublin, Dublin, Ireland). The phylogenetic tree was obtained using MEGA 7.0 software (Institute for Genomics and Evolutionary Medicine, Temple University, Tempe, AZ, USA).

**Figure 2:**
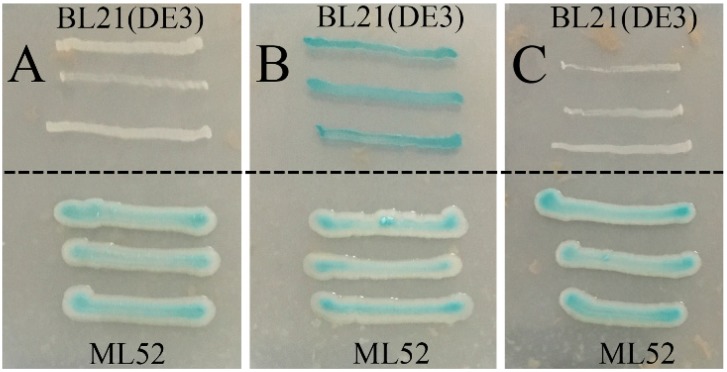
Effects of glucose and lactose on the expression of β-galactosidase of strain ML52. *E. coli* strain BL21(DE3) was used as a control. A, 2216E X-Gal agar with 2% glucose; B, 2216E X-Gal agar with 2% lactose; C, 2216E X-Gal agar with 2% glucose and lactose.

Strain ML52 was isolated from deep-sea water in the Mariana Trench at a depth of 4000 m and found to produce intracellular β-galactosidase at 4 °C. Database searches showed that strain ML52 is related to the genus *Alteromonas*. As shown in the neighbor-joining tree (Figure 1) [34,35], strain ML52 formed a monophyletic cluster with *Alteromonas addita* R10SW13^T^ (99.9% identity), *Alteromonas stellipolaris* LMG 21861^T^ (99.8% identity) and *Alteromonas naphthalenivorans* SN2^T^ (99.3% identity). Strain ML52 was able to produce β-galactosidase on 2216E X-Gal agar in the presence of either lactose or glucose (Figure 2), while the expression of β-galactosidase in *E. coli* containing a lac operon was repressed by glucose [36].

### 2.2. Molecular Cloning and Sequence Analysis

**Figure 3:**
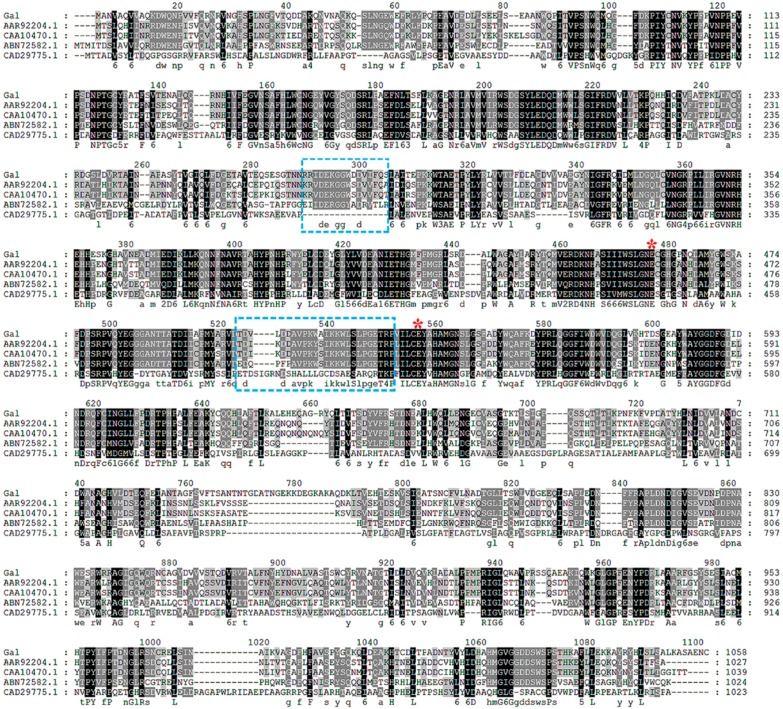
Sequence alignment of Gal and other GH2 β-galactosidase from different microorganisms. Identical residues are shaded in black and conserved residues are shaded in gray. The putative nucleophilic and catalytic amino acids are marked by a red asterisk and the regions relative to the formation of a tetramer are marked by blue boxes. Protein accession numbers and species are as follows: AAR92204.1, *Pseudoalteromonas* sp. 22b; CAA10470.1, *Pseudoalteromonas haloplanktis*; ABN72582.1, *E. coli* K-12; CAD29775.1, *Arthrobacter* sp. C2-2.

Deoxyribonucleic acid sequencing showed that *gal* consisted of an open reading frame of 3174 bp, encoding 1058 amino acid residues. The theoretical Mw and pI of the enzyme was 118,543 Da and 4.96, respectively. According to the BLAST results, *gal* had a highest identity of 99% to a putative β-galactosidase from *Alteromonas addita* (WP_062085674.1). At the time of writing, two characterized β-galactosidase genes from *Pseudoalteromonas* sp. 22b (AAR92204.1) and *Pseudoalteromonas haloplanktis* (CAA10470.1) exhibited the highest sequence identity (65%) to *gal*. Based on sequence comparisons, Gal was classified into glycoside hydrolase family 2. Amino acid sequence comparison of Gal and other characterized GH2 β-galactosidases are shown in Figure 3. 

### 2.3. Expression and Purification of Recombinant Gal

**Figure 4:**
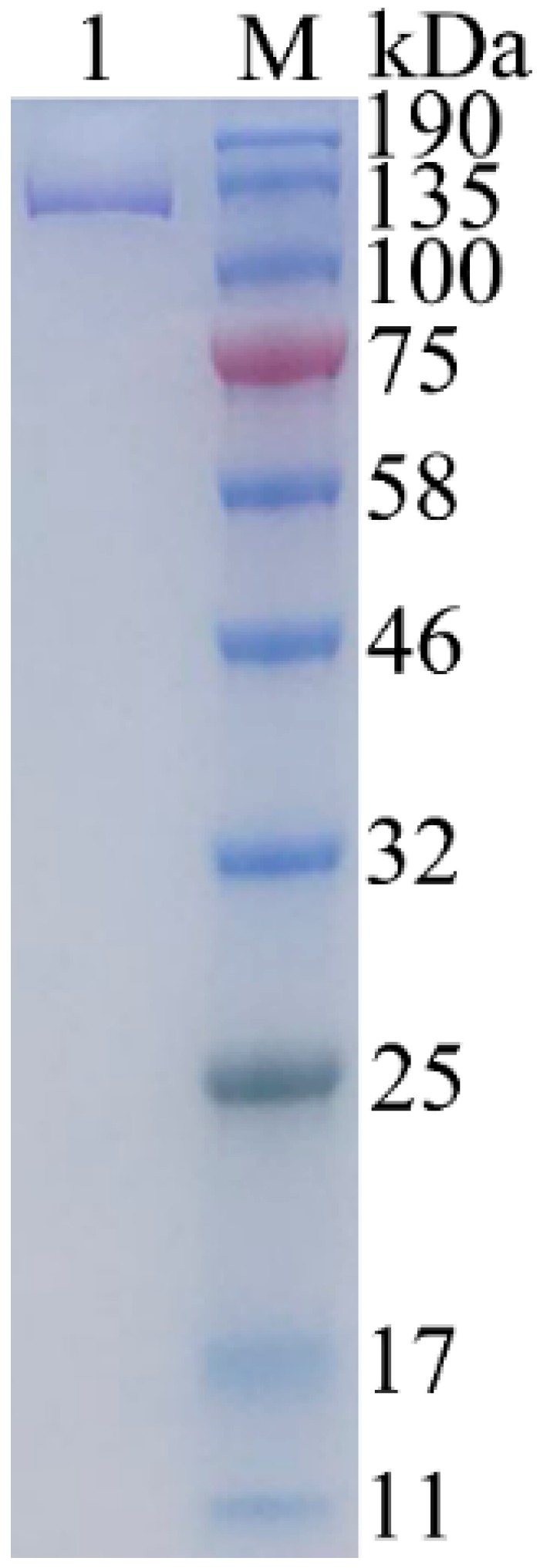
Twelve percent SDS-PAGE analysis of the purified β-galactosidase. Lane 1, purified β-galactosidase; lane M, protein marker. The gel was stained with 0.025% Coomassie blue R250.

The expression vector pET-24a was used for the expression of the *gal* gene from strain ML52 in *E. coli* BL21 (DE3). The recombinant enzyme with a 6-histidine tag was induced by IPTG and purified by Ni-NTA chromatography. After purification, approximately 55.2 mg of pure enzyme was obtained from 1L of induced culture. The apparent molecular weight of recombinant Gal was 126 kDa which was determined by SDS-PAGE (Figure 4), and which corresponded to the theoretical size. The relative molecular weight of recombinant Gal, which was determined by gel-filtration chromatography, was 485 kDa (results not shown). Hence recombinant Gal is probably a tetrameric protein, like *E. coli* β-galactosidase.

### 2.4. Properties of Recombinant Gal

**Figure 5:**
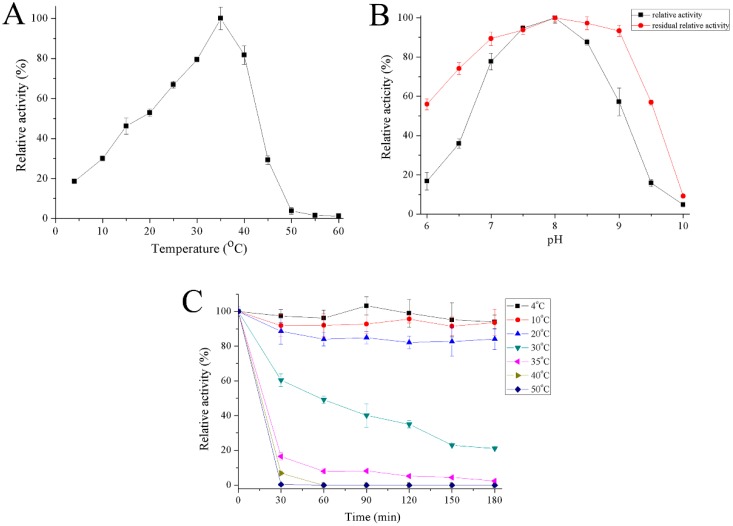
Effect of temperature on activity (**A**) and stability (**C**) of recombinant Gal and effect of pH (**B**) on activity of recombinant Gal. The optimum activity was set as 100% with specific activities of 434.3 U/mg, or 286 U/mg for the effect of temperature or pH, respectively.

The optimum temperature for recombinant Gal was close to 35 °C and the enzyme had 19–30% activity at 4–10 °C (Figure 5A). Recombinant Gal maintained high activity over a pH range of 7 to 8.5 with maximum activity at pH 8. The enzyme was stable from a pH range between 6.5 and 9 (Figure 5B). Recombinant Gal was stable at temperatures between 4 and 20 °C, but its activity reduced rapidly at 30 °C and most activity was lost at 50 °C following half hour incubation (Figure 5C).

**Figure 6:**
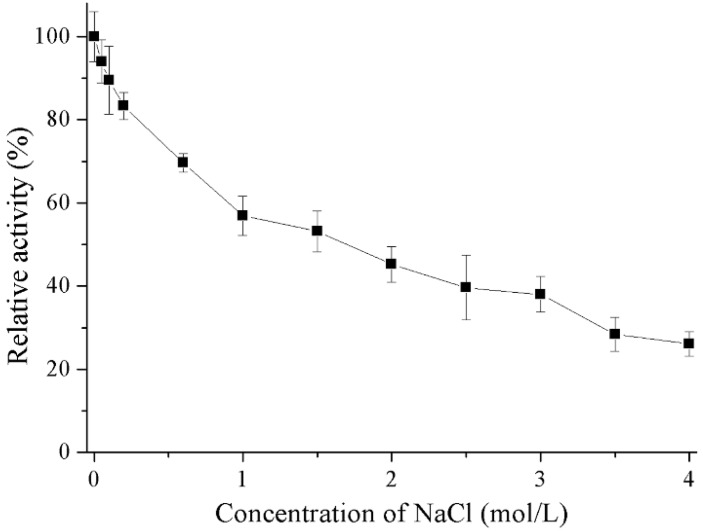
Effects of NaCl on the activity of recombinant Gal. Activity with 0 M NaCl was set as 100% with a specific activity of 445.3 U/mg.

Results outlining the effects of metal ions and chemicals on recombinant Gal are shown in Table 1. Reducing agents (DTT and 2-mercaptoethanol) and K^+^ could stimulated the enzymatic activity of recombinant Gal. The addition of Ca^2+^ and urea slightly decreased enzyme activity whereas Mg^2+^, Mn^2+^ and ionic detergent (SDS) had strong inhibitory effects. The presence of chelating agent (EDTA) and the ions Zn^2+^, Ni^2+^, and Cu^2+^ completely inhibited enzyme activity. Salt tolerance of the recombinant Gal was also investigated (Figure 6). Recombinant Gal maintained 53% residue activity in the presence of 1.5 M NaCl and 26% residue activity when NaCl concentration increased to 4 M.

The substrate specificity of recombinant Gal was investigated using seven chromogenic substrates (Table 2). The enzyme was specific to two β-d-galactopyranoside substrates, and showed no activity to other tested substrates. Kinetic parameters of recombinant Gal were determined at optimum temperature with ONPG (2-Nitrophenyl-β-D-galactopyranoside) and lactose as substrates, as shown in Table 3.

### 2.5. Hydrolysis of Lactose in Milk

**Figure 7:**
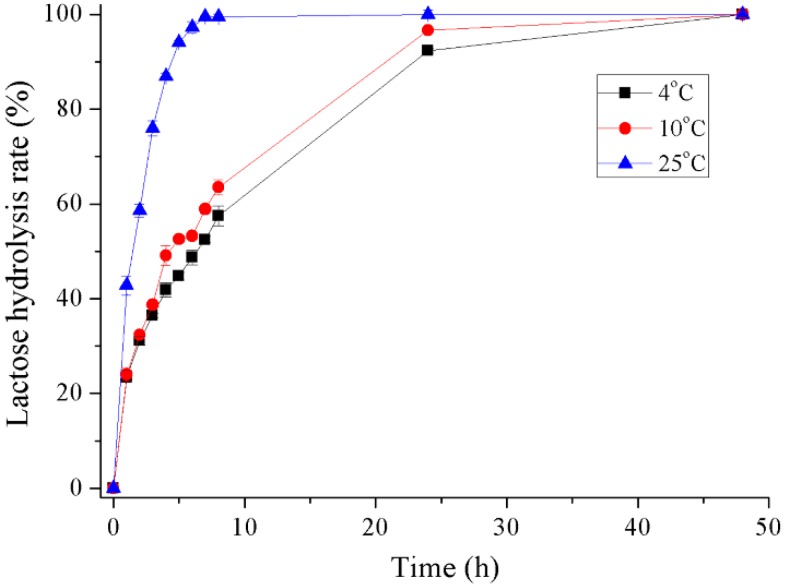
Hydrolysis of lactose in milk of recombinant Gal.

The hydrolysis of milk lactose by recombinant Gal was determined at 4 °C, 10 °C and 25 °C (Figure 7 and Figure S1). The conversion rate of lactose in milk reached about 42% during the initial hour at 25 °C and 94% after 5 h incubation. At refrigerating temperatures of 4 °C and 10 °C, 58% and 64% of lactose was hydrolyzed after 8 h incubation, respectively. A lactose conversion rate of greater than 90% was reached after 24 h incubation and almost 100% after 48 h.

## 3. Discussion

A deep-sea bacterium *Alteromonas* sp. ML52 with β-galactosidase activity was obtained through plate screening. The β-galactosidase from strain ML52 was considered to be a constitutive enzyme because its expression was normal without lactose and not repressed by glucose. A novel GH2 β-galactosidase gene *gal* was cloned from strain ML52. Sequence alignment of Gal and other GH2 β-galactosidases (Figure 2) showed that Gal shares a conserved acid-base activity site and nucleophilic site typically found in this family of enzyme. Compared with a *E. coli* β-galactosidase, a mesophilic enzyme belonging to the GH2 family, the amino acid sequence composition of Gal showed significant decreases in Arg residues (4.5% versus 6.4%), Arg/Arg+Lys ratio (0.51 versus 0.77) and Pro residues (4.9% versus 6.1%) which are structural features of psychrophilic enzymes [30]. Skálová et al. [31] compared two crystal structures of GH2 β-galactosidases, a tetrameric enzyme from *E. coli* and a hexameric enzyme from *Arthrobacter* sp. C2-2, and suggested that the outstanding loop region of domain 2, which participates in forming the contacts between subunits of the tetramer along with domain 3 in *E. coli* β-galactosidase; however, this kind of complementation does not occur in *Arthrobacter* sp. C2-2. The sequences relative to the formation of the tetramer of Gal in domain 2 and domain 3 were similar to that of the *E. coli* β-galactosidase, which are labeled in Figure 3. This theoretical prediction was tested by molecular weight determination. Thus, Gal is considered to be a homotetrameric protein.

Recombinant Gal has an optimum temperature of 35 °C and a low thermal stability over 30 °C. Other reported GH2 cold-adapted β-galactosidases possessed optimum temperatures between 10 and 40 °C (Table 4). Thus Gal, which was isolated from a deep-sea bacterium, is considered to be a cold-adapted enzyme. The optimum pH range for enzyme activity and stability was consistent with bacterial β-galactosidases which are normally active within a neutral pH range of 6 to 8. This feature indicates that Gal may be a suitable enzyme for lactose hydrolysis in milk (pH 6.5–6.8). The activating effect of K^+^ and the inhibitory action of ions Zn^2+^, Ni^2+^, Cu^2+^ to the enzyme activity of recombinant Gal also occurred in a cold-adapted *Pseudoalteromonas* sp. 22b β-galactosidase [28], with the highest sequence identity to Gal. The chelating agent caused a complete inhibition of enzyme activity, indicating that the catalytic reaction of recombinant Gal may rely on the presence of metal ions. It was also noted that recombinant Gal preferred substrate ONPG to substrate PNPG. This phenomenon was also observed in cold-adapted β-galactosidases from *Paracoccus* sp. 32d [23] and *Arthrobacter psychrolactophilus* strain F2 [20], but many cold-adapted β-galactosidases, isolated from *Pseudoalteromonas* sp. 22b [28], *Arthrobacter* sp. 20B [37] and *Arthrobacter* sp. 32c [21], displayed higher levels of activity to PNPG. Remarkably, recombinant Gal showed significantly high affinity and reaction rate to the chromogenic substrate ONPG at optimum temperature compared to other GH2 cold-adapted β-galactosidases from *Arthrobacter psychrolactophilus* strain F2, *Arthrobacter* sp. and *Paracoccus* sp. 32d (Table 4). This property is similar to the *Pseudoalteromonas* sp. 22b β-galactosidase. For lactose, the natural substrate of β-galactosidase, recombinant Gal showed intermediate substrate affinity and catalytic efficiency at optimum temperature. The major industry β-galactosidases were obtained from *Aspergillus* spp. and *Kluyveromyces* spp. [1,4,8]. The β-galactosidases from *Aspergillus* spp. have an optimum pH at acidic range (2.5–5.4) and are not suitable for hydrolysis of lactose in milk. The β-galactosidase from *Kluyveromyces lactis* is one of the most widely used commercial enzymes. The optimum pH (6.6–7) of *K. lactis* β-galactosidase is close to milk because the dairy environment is a natural habitat for this kind of yeast [4]. However, *K. lactis* β-galactosidase showed higher optimum temperature (40 °C) [38] and lower substrate affinity to both ONPG and lactose (*K*_m_, 1.7 mM for ONPG and 17.3 mM for lactose) [8] compared with recombinant Gal.

The results on lactose digestion in milk indicate that recombinant Gal could hydrolyze 100% of lactose in milk when incubated for 7 h at 25 °C and over 90% lactose was hydrolyzed after incubation for 24 h at 4 °C and 10 °C. No transglycosylation product was observed by HPLC during the hydrolysis process. For the *Pseudoalteromonas* sp. 22b cold-adapted β-galactosidase [28], 90% of lactose was hydrolyzed after 6 h at 30 °C and after 28 h at 15 °C. Another cold-adapted β-galactosidase from the *Arthrobacter psychrolactophilus* strain F2 [20] was able to hydrolyze approximately 70% of the lactose in milk at 10 °C after 24 h and displayed transglycosylation activity by forming a trisaccharide product during the reaction.

## 4. Materials and Methods

### 4.1. Isolation and Identification of Bacteria

Deep-sea water samples were collected from the Mariana Trench at a depth of 4000 m in September 2016. They were diluted and spread on marine agar 2216E (MA; BD Difco, Franklin Lakes, NJ, USA) containing 40 μg/mL X-Gal (5-bromo-4-chloro-3-indolyl-β-d-galactopyranoside, Sigma, St. Louis, MO, USA) and 2% lactose. After incubation at 4 °C for 2 weeks, the detectable blue colonies were repeatedly streaked on MA to obtain pure cultures. The 16S rRNA gene of strain ML52 was amplified by PCR from genomic DNA. To test the effects of glucose and lactose on the expression of β-galactosidase, strains were grown on MA containing 40 μg/mL X-gal and 2% glucose or lactose at 25 °C for 2 days.

### 4.2. Molecular Cloning and Sequence Analysis

Based on the DNA sequence of the predicted β-galactosidase gene (Accession number AMJ93096.1) from *Alteromonas addita* strain R10SW13 (Accession number CP014322.1) reported in the NCBI Database, two primers, forward primer-CG*GGATCC*ATGGCAAATGTTGCTCAA and reverse primer-CCG*CTCGAG*GCAATTTTCAGCACT (*Bam*HI and *Xho*I restriction sites are in italics) were designed. The PCR product was cloned into pMD20-T and sequenced by Tsingke, China. The plasmid pET-24a and amplified *gal* gene were then cleaved with the restriction endonucleases *Bam*HI and *Xho*I and the fragments were ligated with T4 DNA ligase (NEB), resulting in the plasmid pET-*gal*. The nucleotide sequence of *gal* was translated to an amino acid sequence using the ExPASY-Translate tool. A multiple alignment between Gal and other GH2 β-galactosidases from bacteria was constructed using ClustalW 2 (Conway Institute UCD Dublin, Dublin, Ireland) and GeneDoc 2.7 (Karl B. Nicholas et al., San Francisco, CA, USA). The theoretical molecular weight (Mw) and isoelectric point (pI) were calculated using the Compute pI/Mw tool (http://web.expasy.org/compute_pi/).

### 4.3. Expression and Purification

For expression of recombinant Gal, the plasmid pET-*gal* was transformed into *E. coli* BL21 (DE3) and cultivated at 37 °C in lysogeny broth (LB) containing 30 μg/mL kanamycin until the OD_600_ of the culture reached 0.6–0.8. Following this step, isopropyl-β-d-thiogalactopyranoside (IPTG) was added to the culture with a final concentration of 0.2 mM and the culture was further grown at 16 °C for 12 h. The cells were harvested by centrifugation at 8000 rpm for 10 min at 4 °C and suspended in a lysis solution (50 mM sodium phosphate buffer, pH 8). The mixture was then disrupted by sonication and the cell debris removed by centrifugation at 13,000 rpm for 15 min. The filtered supernatant was applied to a Ni^2+^-chelating affinity column (His-Trap^TM^ HP, GE, Madison, WI, USA) which was previously equilibrated using an equilibration buffer (50 mM sodium phosphate buffer, 20 mM imidazole, 300 mM NaCl, pH 8) and subsequently eluted using a linear gradient of 20–250 mM imidazole in equilibration buffer. Enzyme purity was analyzed by SDS-PAGE (GenScript, Nanjing, China). Purified recombinant Gal was buffer-exchanged to 50 mM sodium phosphate buffer (pH 8) via centrifugal ultrafiltration (MW cut off 10 kDa, Millipore, Burlington, MA, USA). The protein concentration was determined using the BCA protein assay kit (Solarbio, Beijing, China) with bovine serum albumin as a standard.

### 4.4. Estimation of Molecular Weight

The purified enzyme was applied to a gel-filtration column (Superdex 200 HR 10/30, GE Healthcare, Madison, WI, USA) and eluted using a buffer containing 50 mM sodium phosphate (pH 7) and 150 mM NaCl. The standard proteins used were thyroglobumin (669 kDa), apoferritin (443 kDa), β-amylase (200 kDa), alcohol dehydrogenase (150 kDa), bovine serum albumin (66 kDa), and carbonic anhydrase (29 kDa) purchased from Sigma (St. Louis, MO, USA).

### 4.5. Beta-Galactosidase Activity Assay

Beta-galactosidase activity was determined in a 100 μL reaction mixture containing a final concentration of 0.4 ng/μL (0.83 nM) of purified recombinant Gal, 50 mM sodium phosphate buffer (pH 8) and 5 mM 2-nitrophenyl-β-d-galactopyranoside (ONPG). Each reaction was incubated for 5 min at 35 °C and quenched by the addition of 200 μL of 1 M Na_2_CO_3_. The absorbance of the released o-nitrophenol (ONP) was measured at 420 nm and quantified using an ONP standard curve. One unit (U) of enzyme activity was defined as the amount of enzyme required for the liberation of 1 µmol ONP per minute under the assay conditions. All assays were carried out in triplicate.

### 4.6. Effect of Temperature and pH

The optimum temperature of recombinant Gal was evaluated by incubating the reaction mixtures at different temperatures ranging from 4 °C to 60 °C. The optimum pH was determined by incubation in 10 mM Britton–Robinson buffers ranging from pH 6 to 10 (in increments of 0.5 pH units) at optimum temperature. The thermostability of recombinant Gal was determined by incubating enzymes at temperatures ranging from 4 °C to 50 °C for 180 min and the residual enzyme activity was determined every 30 min under the optimum conditions. The pH stability was determined after incubating the enzyme in Britton–Robinson buffers ranging from pH 6 to 10 (in increments of 0.5 pH units) at 4 °C for 3 h.

### 4.7. Effect of Metal Ions and Chemicals

The effect of metal ions and chemicals on recombinant Gal activity was determined after incubating the enzyme in water with 5 mM of KCl, CaCl_2_, MgCl_2_, MnCl_2_, BaCl_2_, ZnCl_2_, NiSO_4_, CuCl_2_, EDTA, urea, DTT, 2-mercaptoethanol or SDS at 4 °C for 1 h. The activity of the enzyme when incubated without any additional reagents was considered to be 100%. For salt stability, 0–4 M NaCl (final concentration) was added to the reaction mixtures and the activity determined using optimum conditions.

### 4.8. Determination of Substrate Specificity and Kinetic Parameters

The substrate specificity of the recombinant Gal was estimated using 5 mM (final concentration) ONPG, 4-Nitrophenyl-β-d-galactopyranoside (PNPG), 4-Nitrophenyl-α-d-galactopyranoside, 2-Nitrophenyl-β-d-glucopyranoside, 4-Nitrophenyl-β-d-glucopyranoside, 4-Nitrophenyl-α-d-glucopyranoside or 4-Nitrophenyl-β-d-xylopyranoside in 50 mM sodium phosphate buffer (pH 8) under the optimum conditions. For the activity of 4-nitrophenol-derived substrates, the absorbance of the released 4-nitrophenol (PNP) was measured at 405 nm and quantified using a PNP standard curve. Kinetic parameters of the recombinant Gal were determined at 35 °C and the reaction rate with ONPG (0.1–5 mM) and lactose (0.2–40 mM) as substrates was determined. The released glucose in the lactose hydrolysis reaction was measured using a commercial glucose oxidase-peroxidase assay kit (Shanghai Rongsheng Biotech Co., Ltd., Shanghai, China). One unit (U) of enzyme activity was defined as 1 μmol of glucose released per minute. The values of the kinetic constants were calculated using the Lineweaver-Burk method.

### 4.9. Hydrolysis of Lactose in Milk

The hydrolysis of lactose in milk was determined by incubating 100 μg purified Gal in 1 mL of commercial skim milk (Inner Mongolia Yili Industrial Group Co. Ltd., Hohhot, China) at 4 °C, 10 °C, or 25 °C for 48 h. The reaction was terminated by incubating the sample at 60 °C for 5 min and then adding an equal volume of 5% trichloroacetic acid (TCA) to the reaction mixture. The pH of the reaction mixture was adjusted to neutral pH using 1 M NaOH and centrifuged at 10,000 rpm for 10 min. The supernatant was analyzed by HPLC using the ZORBAX Carbohydrate Analysis Column (Agilent, Palo Alto, CA, USA), with 75% acetonitrile used as a mobile phase at a flow rate of 1 mL/min and a Shimadzu Refractive Index Detector (Kyoto, Japan).

### 4.10. Nucleotide Sequence Accession Numbers

The 16S rRNA gene sequence and β-galactosidase gene (*gal*) of strain ML52 were deposited in GenBank under the accession numbers MH916568 and MH925304, respectively.

## 5. Conclusions

In this study, a new GH2 β-galactosidase (Gal) was successfully cloned, purified and characterized from the deep-sea bacterium *Alteromonas* sp. ML52. Recombinant Gal is a cold-adapted enzyme and was able to efficiently hydrolyze lactose in milk at refrigerating temperature. These characteristics suggest that Gal could be a potential cold-active biocatalyst and usefully applied to the production of lactose-free milk in the dairy industry.

## Figures and Tables

**Table 1:** Effect of metal ions and chemicals on activity of recombinant Gal.

Ion/Reagent	Relative Activity (%)
None ^1^	100 ± 1.3
K^+^	123 ± 0.6
Ca^2+^	74.9 ± 5.4
Mg^2+^	32.4 ± 1.4
Mn^2+^	21.3 ± 0.9
Ba^2+^	48.2 ± 2.1
Zn^2+^	0.1 ± 0.04
Ni^2+^	0.2 ± 0.03
Cu^2+^	0
EDTA	0.2 ± 0.02
Urea	89.9 ± 0.3
DTT	116.9 ± 7
2-Mercaptoethanol	108.9 ± 4
SDS	43.5 ± 1

^1^ The activity of control (no additions) was set as 100% with a specific activity of 386.7 U/mg.

**Table 2:** Substrate specificity of recombinant Gal.

Substrate	Relative Activity (%)
2-Nitrophenyl-β-d-galactopyranoside (ONPG) ^1^	100 ± 4.2
4-Nitrophenyl-β-d-galactopyranoside (PNPG)	12.8 ± 0.5
4-Nitrophenyl-α-d-galactopyranoside	0
2-Nitrophenyl-β-d-glucopyranoside	0
4-Nitrophenyl-β-d-glucopyranoside	0
4-Nitrophenyl-α-d-glucopyranoside	0
4-Nitrophenyl-β-d-xylopyranoside	0

^1^ The activity of ONPG was set as 100% with a specific activity of 467.2 U/mg.

**Table 3:** Substrate specificity of recombinant Gal.

Substrate	*V*_max_ (U/mg)	*K*_m_ (mM)	*k*_cat_ (S^−1^)	*k*_cat_/*K*_m_ (S^−1^ mM^−1^)
ONPG	464.7	0.14	3688.1	26343.6
Lactose	18.5	7.2	146.5	20.3

**Table 4:** Biochemical characteristics of reported GH2 cold-adapted β-galactosidases.

Strain	Optimum		ONPG		Lactose		References
	Temperature (°C)	pH	*K*_m_ (mM)	*k*_cat_ (S^−1^)	*K*_m_ (mM)	*k*_cat_ (S^−1^)	
*Alteromonas* sp. ML52	35	8	0.14	3688.1	7.2	146.5	This work
*Arthrobacter psychrolactophilus* strain F2	10	8	2.7	12.7	42.1	3.02	[20]
*Arthrobacter* sp. 20B	25	6–8	-	-	-	-	[37]
*Arthrobacter* sp. C2-2	40	7.5	-	-	53.1	1106	[39]
*Arthrobacter* sp.	18	7	11.5	5.2	-	-	[17]
*Paracoccus* sp. 32d	40	7.5	1.17	71.81	2.94	43.23	[23]
*Pseudoalteromonas haloplanktis* TAE 79	-	8.5	-	203	2.4	33	[24]
*Pseudoalteromonas* sp. 22b	40	6–8	0.28	312	3.3	157	[28]

## Supplementary Materials

The following are available online at http://www.mdpi.com/1660-3397/16/12/469/s1, Figure S1: HPLC-RID profiles of hydrolysis of lactose in milk at 4 °C (A), 10 °C (B) and 25 °C (C). (1) Glucose; (2) Galactose; (3) Lactose.
