# Supplementary material for: Cloning, Expression and Characterization of a Novel Cold-adapted β-galactosidase from the Deep-sea Bacterium Alteromonas sp. ML52
Source: Mar Drugs. 2018 Nov 27;16(12):469. doi: 10.3390/md16120469 (PMC6315854; doi:10.3390/md16120469)
Supplement: Supplementary file 1 [file marinedrugs-16-00469-s001.pdf]

Supplementary materials for

Cloning, expression and characterization of a novel cold-adapted β-galactosidase from the deep-sea bacterium *Alteromonas* sp. ML52

Jingjing Sun [1,2,*], Congyu Yao [1,3], Wei Wang [1,2], Zhiwei Zhuang [4], Junzhong Liu [1,2], Fangqun Dai [1,2] and Jianhua Hao [1,2,5,*]

1. Key Laboratory of Sustainable Development of Polar Fishery, Ministry of Agriculture and Rural Affairs, Yellow Sea Fisheries Research Institute, Chinese Academy of Fishery Sciences Qingdao 266071, China; sunjj@ysfri.ac.cn (J.S.); yaocongyv@foxmail.com (C.Y.); weiwang@ysfri.ac.cn (W.W.); qdjz99@163.com (J.L.), dai@ysfri.ac.cn (F.D.); haojh@ysfri.ac.cn (J.H.)
2. Laboratory for Marine Drugs and Bioproducts, Laboratory for Marine Fisheries Science and Food Production Processes, Qingdao National Laboratory for Marine Science and Technology Qingdao 266071, China.
3. Shanghai Ocean University, Shanghai 201306, China.
4. New Hope Liuhe Co. Ltd., Qingdao 266071, China; zzw19680@163.com (Z.Z.)
5. Jiangsu Collaborative Innovation Center for Exploitation and Utilization of Marine Biological Resource, Lianyungang 222005, China.

*Correspondence: sunjj@ysfri.ac.cn (J.S.); haojh@ysfri.ac.cn (J.H.); Tel.: +86-532-8584-1193 (J.S.); +86-532-8581-9525 (J.H.)

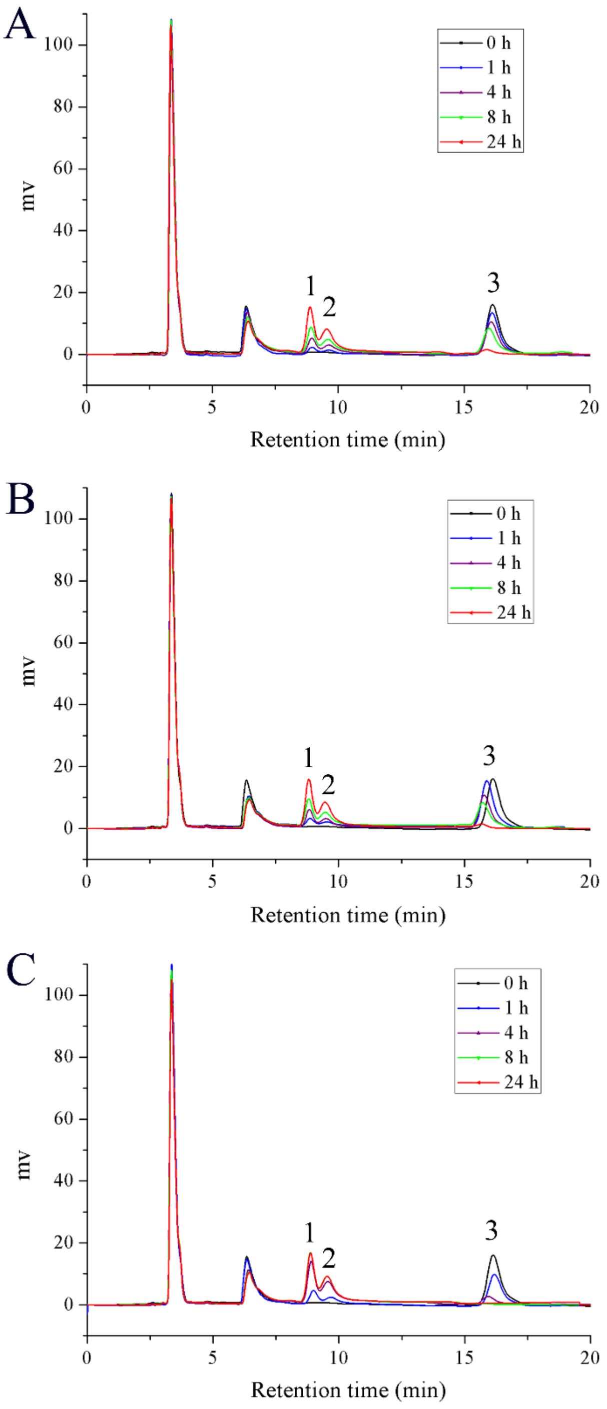

Figure S1. HPLC-RID profiles of hydrolysis of lactose in milk at 4 ℃ (A), 10 ℃ (B) and 25 ℃ (C). (1) Glucose; (2) Galactose; (3) Lactose.
